# Goats Naturally Infected with the Spanish Goat Encephalitis Virus (SGEV): Pathological Features and An Outbreak

**DOI:** 10.3390/ani13010072

**Published:** 2022-12-24

**Authors:** Ana Balseiro, Claudia Pérez-Martínez, Mark P. Dagleish, Luis J. Royo, Laura Polledo, Juan F. García Marín

**Affiliations:** 1 Departamento de Sanidad Animal, Facultad de Veterinaria, Universidad de León, 24071 León, Spain; 2 Departamento de Sanidad Animal, Instituto de Ganadería de Montaña (CSIC-Universidad de León), Finca Marzanas, Grulleros, 24346 León, Spain; 3 Pathology Department, School of Biodiversity, One Health and Veterinary Medicine, University of Glasgow, Glasgow G12 8QQ, Scotland, UK; 4 Departamento de Biología Funcional, Genética, Universidad de Oviedo, 33006 Oviedo, Spain

**Keywords:** Spanish goat encephalitis virus (SGEV), goat, outbreak, histopathology, immunohistochemistry

## Abstract

**Simple Summary:**

The aims of the present study were to describe the pathology in goats naturally infected with the Spanish goat encephalitis virus (SGEV), as well as discuss the pathogenesis of the disease in the outbreak which occurred in 2011. Neuropathological lesions caused by SGEV were severe and widespread throughout the central nervous system but were more severe and extensive in the proximal cervical spinal cord, medulla oblongata, pons, and cerebellar cortex. The distribution of viral antigens was restricted to the cytoplasm of neurons in several brain areas but not associated with inflammatory foci nor inflammatory cells. SGEV should be considered a significant pathogen of goats that results in severe neurological clinical disease and high mortality.

**Abstract:**

In autumn 2011, a disease outbreak caused by Spanish goat encephalitis virus (SGEV) was reported in a herd of goats from Asturias (north-western Spain), expanding the known geographic distribution of tick-borne encephalitis in Europe. The virus was classified as a new subtype (subspecies) within the Louping-ill virus species of the mammalian tick-borne flavivirus group. The aims of the present study were to describe the pathology in goats naturally infected with SGEV, as well as discuss the pathogenesis of the disease in that outbreak. A total of 22/85 (25.88%) goats (20 adults and 2 kids) died between October 2011 and June 2012, showing neurological clinical signs. Over three years, the mortality rate in the herd reached 100%. Neuropathological lesions caused by SGEV were severe and widespread throughout the central nervous system but were more severe and numerous in the proximal cervical spinal cord, medulla oblongata, pons and cerebellar cortex. They consisted of neuron necrosis, neuronophagia, mononuclear inflammatory cell perivascular cuffs (lymphocytes, plasma cells and macrophages) and gliosis. The distribution of viral antigens was restricted to the cytoplasm of neurons in several brain areas but not associated with inflammatory foci nor inflammatory cells. SGEV should be considered a significant pathogen of goats that results in severe neurological clinical disease and high mortality.

## 1. Introduction

Human- and animal-shared infectious diseases, such as vector-borne diseases, are emerging continually and also spreading to new geographic locations influenced by human encroachment and environmental conditions (e.g. climate change) [1]. Louping-ill (LI) is an acute zoonotic viral disease of the central nervous system caused by the louping-ill virus (LIV), a member of the genus *Flavivirus* that belongs to the tick-borne encephalitis (TBE) group of viruses [1,2]. It includes several subtypes, including British, Irish, Turkish and Spanish variants. The current taxonomic classification, specified by the International Committee on Taxonomy of Viruses, states that these viruses are all subtypes (subspecies/variants) within the LIV species of mammalian tick-borne flaviviruses, apart from tick-borne encephalitis virus (TBEV), which is designated a separate species [2]. Ticks within the genus *Ixodes* are considered the natural vectors of LIV, but other genera (*Dermacentor* and *Haemaphysalis*) can also be involved [3]. Although many domestic (goats, cattle, horses, pigs and dogs) and wildlife species (several deer species, llamas and monkeys) may become infected with LIV, only sheep and red grouse (*Lagopus lagopus scotica*) are believed to develop a viraemia sufficient to infect ticks [3,4,5]. Louping-ill is endemic in rough upland areas in Scotland, England, Wales and Ireland [4], and in Spain, the presence of TBE viruses causing LI-like disease, i.e. Spanish sheep encephalomyelitis virus (SSEV), was reported in the Basque Country (north Spain) in the 1980s [6]. In autumn 2011, an outbreak of LI-like disease was reported in a herd of goats from Asturias (north-western Spain) [7], increasing the known geographic distribution and host species of the TBE group within Europe. Despite the proximity of the Basque country to Asturias (400 km), whole genome sequencing and phylogenetic analysis of the virus isolated demonstrated that the genome sequence diverged significantly from those of LIV and SSEV. This novel virus encoded an amino acid sequence motif shared with a virus isolated in Ireland in 1968 [8]. The virus was classified as a new subtype (subspecies) within the LIV species of the mammalian tick-borne flavivirus group and named Spanish goat encephalitis virus (SGEV) to distinguish it from SSEV [8].

Few reports of LI in goats exist, either in experimentally [9,10] or naturally acquired [11,12] infections, and a complete description of lesions in goats caused by naturally acquired SEGV has not been reported previously. The aims of the present study were to: (1) describe fully the histological lesions of naturally acquired SGEV in goats from the 2011 outbreak; (2) determine the cell tropisms and relationship between the presence of the viral antigens and the type and distribution of lesions by means of specific immunohistochemistry (IHC); and (3) discuss the pathogenesis of the disease in this outbreak.

## 2. Materials and Methods

### 2.1. Goat Herd History

In September 2011, a herd of 70 adult goats (2 males/68 females) was purchased in Allande (43°26′67″ N 6°61′66″ W, Asturias, Spain) and translocated 120 km to Quirós (43°12′17″ N 6°1′6″ W, Asturias, Spain), where fifteen kids (7 females/8 males) were born on the farm over the following two months. A total of 22/85 (25.88%) goats (20 adults and 2 kids) died between October 2011 and June 2012. The first neurological clinical signs, which consisted of depression, hyperaesthesia, ataxia, muscular incoordination, posterior paralysis, tremors and coma, appeared in three goats in October 2011. Over the ensuing four months, 17 adult goats and 2 kids (all females) died prior to the herd being vaccinated by a single dose of an inactivated Louping-ill vaccine (Intervet, Milton Keynes, United Kingdom) on 25 January, 2012. During the following two months, no further mortalities occurred. Subsequently, three more goats died, the last in June 2012. In the three following years all the remaining goats died after developing similar neurological clinical signs, except for a small number of kids that were predated, and the farmer subsequently abandoned goat farming. The etiological agent of the outbreak was identified as SGEV [7,8].

### 2.2. Pathological Examination

Three adult goats were subjected to a necropsy. Goats 1 and 2 died in November 2011 and goat 3 in May 2012, approximately three months after the herd was vaccinated. The goats died 7 (Nos. 1 and 2) or 3 (No. 3) days after the onset of clinical signs. Samples for histological examination included whole brain, proximal cervical spinal cord, lungs, kidney, liver, spleen and gastro-intestinal tract (oesophagus, rumen, omasum, abomasum, small and large intestine), which were fixed in 10% neutral buffered formalin. Afterwards, the brain was sliced coronally to obtain tissue samples of the frontal, parietal and occipital lobes, corpus callosum, thalamus and hypothalamus, midbrain, pons, cerebellum and medulla oblongata. Tissue samples were processed routinely through graded alcohols prior to embedding in paraffin wax. Sections (4 μm) from each block were mounted on separate glass microscope slides and stained with hematoxylin and eosin (HE). Semi-serial sections (3 μm) of the neurological tissue were also subjected to IHC specific for LIV antigens as reported previously, with minor modifications [13]. This antibody had been proven to cross-react with SGEV previously during an experimental challenge [10]. Specifically, the conjugated secondary antibody was visualized by the addition of Vector NovaRED (Vector Laboratories, Peterborough, United Kingdom) for 10 minutes to provide a red rather than brown pigment. Negative control sections consisted of semi-serial sections of all tissues with the primary antibodies substituted by isotype-matched mouse antibodies.

## 3. Results

Necropsy confirmed the presence of several ticks from the genus *Haemaphysalis* on the carcasses. Other than congestion of meningeal vessels, no gross lesions were observed in any of the three animals. All three cases were characterized histologically by severe non-suppurative encephalomyelitis. Lesions were consistently more severe and widespread in the cervical spinal cord, brainstem (medulla oblongata, pons and mid-brain) and cerebellar cortex than in the other anatomical locations of the central nervous system examined. Lesions consisted primarily of: (1) perivascular cuffs comprised of mononuclear inflammatory cells; (2) diffuse and/or focal proliferation of glial cells; and (3) neuron necrosis and neuronophagia. Histological lesions and IHC results from each goat are presented in Table 1.

**Figure 1:**
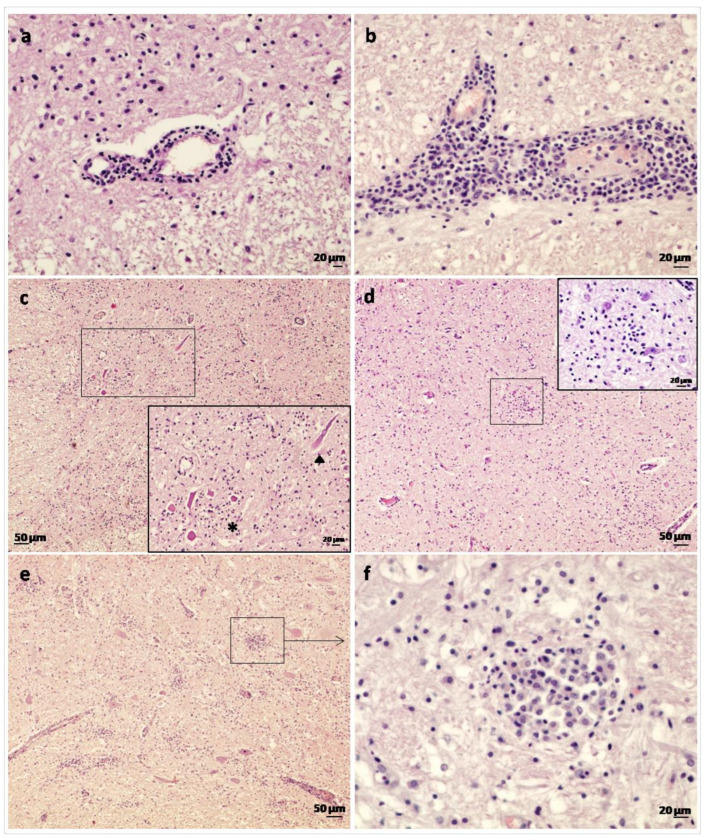
Histological lesions in goats affected by the Spanish goat encephalitis virus (SGEV) as shown by HE staining. (**a**) Spinal cord: thin perivascular cuff comprising only one to two layers of small mononuclear inflammatory cells. (**b**) Midbrain: thick perivascular cuff which consisted of lymphocytes, plasma cells and macrophages. (**c**) Spinal cord: widespread gliosis in grey matter. Inset: Area containing several necrotic neurons (*) and axonal tumefaction (arrowhead). (**d**) Midbrain: occasional glial focus present. Inset: Detail of glial focus composed of microglial cells. (**e**) Cerebellar peduncles: severe widespread inflammation and multiple glial foci in the vaccinated goat (No. 3). (**f**) Cerebellar peduncles: detail of a glial focus comprised primarily of macrophages with fewer numbers of lymphocytes and glial cells.

In the spinal cord, medulla oblongata and pons, perivascular cuffs were widespread and composed mostly of a single or double layer of mononuclear inflammatory cells (Figure 1a). In the thalamus, hypothalamus, cerebellar peduncles, midbrain and corpus callosum, the perivascular cuffs were less numerous but consisted of many layers of lymphocytes, plasma cells and macrophages (Figure 1b). Perivascular cuffing in goat No. 3, which had been vaccinated, was more severe than in the non-vaccinated goats (Nos. 1 and 2) in terms of both the amount of cellular inflammation and distribution. In all three goats examined, the cortical regions of the cerebrum showed occasional perivascular cuffs comprising only a single layer of mononuclear inflammatory cells. Foci of gliosis (composed mainly of microglia and astrocytes) were widespread within the proximal cervical spinal cord (affecting both white and grey matter, and both dorsal and ventral horns), medulla oblongata, pons, cerebellar peduncles, thalamus and hypothalamus. In goat No. 2, diffuse gliosis was observed in the spinal cord (Figure 1c). In the midbrain, gliosis was more diffuse and only occasional small glial foci were observed (Figure 1d). In the frontal lobe of the cerebral cortex, occasional glial foci were present in the border between the corona radiata and cortical grey matter. In goat No. 3, cellular inflammation was much more severe compared to the non-vaccinated goats as denoted by numerous inflammatory foci consisting of macrophages, lymphocytes and glial cells (Figure 1e,f) that were most frequently located in the cerebellar peduncles. Lymphocytes were present in the choroid plexus of goat No. 1. Neuron necrosis and neuronophagia were present consistently in all 3 goats, with features including pyknosis (condensation of the chromatin in the nucleus), chromatolysis, axonal swelling or tumefaction, atrophy, degeneration, and cell lysis (Figure 1c).

**Figure 2:**
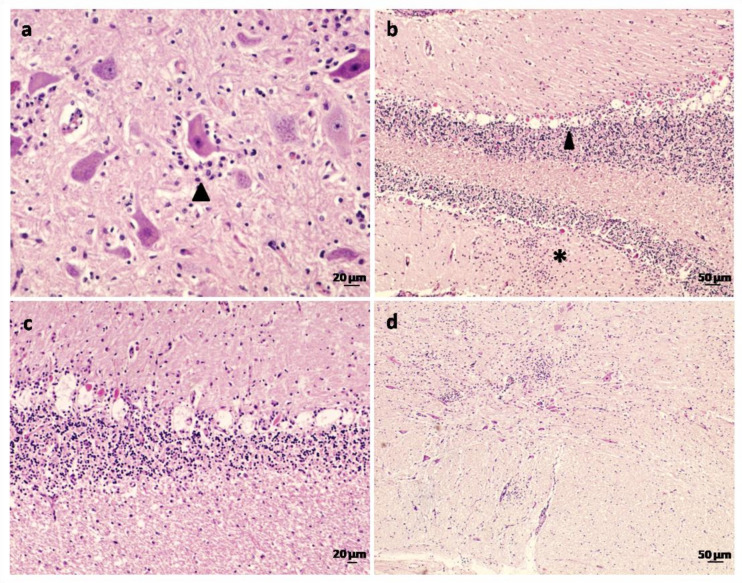
Histopathological lesions in goats affected by Spanish goat encephalitis virus (SGEV) as shown by HE staining. (**a**) Midbrain: neuronophagia—neurons showing atrophy and satellite cells (arrowhead). (**b**) Cerebellar cortex: loss of Purkinje cells (arrowhead) and proliferation of Bergmann cells within the molecular layer (*). (**c**) Cerebellum: detail of the depletion and degeneration of Purkinje cells, in some cases marked by empty spaces (Purkinje cell ‘drop-out’). (**d**) Medial accessory olivary nucleus: multiple foci of gliosis and loss of neurons.

Necrotic neurons were present throughout the brain but less common in the cerebral cortex. The presence of satellite cells secondary to neuronal degeneration and neuronophagia was common (Figure 2a). Purkinje cells were variably affected, and changes varied from degeneration of a few cells to widespread necrosis and depletion affecting the majority of the cerebellar folia (Figure 2b). In such cases, there was often proliferation of Bergmann glial cells (Figure 2b) and, in some instances, the loss of Purkinje cells was marked by empty spaces (Figure 2c). Loss of neurons was found in several nuclei including the red, olivary, medial accessory olivary, reticular formation and nuclei of the vagal and hypoglossal nerves (Figure 2d). Inclusion bodies were not observed. No relevant lesions were found in the other tissues studied, i.e., lungs, kidney, liver, spleen or any of the intestinal samples examined.

**Figure 3:**
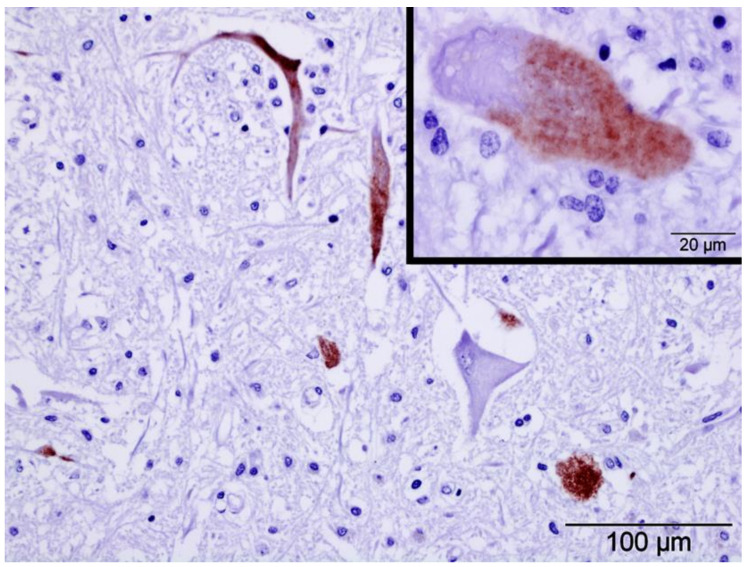
Immunohistochemical preparations in the ventro-lateral medulla oblongata of goat No. 2 using anti-louping-ill virus antibodies. Note positive immunolabelling (red pigment) within the cytoplasm of neurons; positive immunolabelling was rarely associated with inflammatory cells and adjacent neurons could be devoid of labelling. Inset: Intracytoplasmic immunolabelling of a large neuron from the midbrain of the same animal. Both preparations were counterstained with haematoxylin.

Specific IHC for LIV antigens showed positive immunolabelling in the pons and medulla oblongata from goat No. 1 and the midbrain, pons, cerebellum, medulla oblongata and spinal cord from goat No. 2. Labelling was intra-cytoplasmic, restricted to neurons, primarily large neurons, all of which were not associated with inflammatory cells (Figure 3). No immunolabelling was detected in the samples from goat No. 3. All negative control preparations were devoid of any immunolabelling.

## 4. Discussion

Naturally acquired infections by any of the other viruses within the LIV species (LIV, SSEV) causing clinical disease in adult goats and resulting in severe encephalomyelitis has not been reported previously. Histological examination of the brain of an adult goat that died in Scotland due to natural infection with LIV showed only chronic low-grade lesions [12]. Furthermore, the brains of adult goats infected experimentally with LIV showed only mild non-suppurative meningoencephalitis, although the lesions were more severe in one goat that developed clinical signs [9]. However, severe encephalitis in goat kids infected with LIV experimentally, in the United Kingdom [9], and naturally, in Greece [11], has been described. Field infection with SGEV, as occurred in this goat herd, caused severe, acute, non-suppurative encephalomyelitis and, over the time course of the whole outbreak, resulted in a mortality rate of virtually 100% based on clinical signs of the goats that died after the initial extensive investigation. Previous disease outbreaks in sheep or goats due to LIV or SSEV, both closely related viruses, have never reached such a high mortality rate and this suggests that the virulence of SGEV in goats to this, possibly host-adapted, flavivirus, is much greater. Experimental infections with SGEV in goat kids and lambs resulted in both a lower incidence of clinical disease and less severe histological lesions [10,14]. Additionally, these experimental infections did not result in deaths of any goat kids or lambs, although several factors, such as lack of other stressors, were proposed as a possible explanation for the lower morbidity, milder lesions and absence of mortality.

To determine exposure to viruses from the TBEV family in the specific region, a serological survey was undertaken in 178 adult goats, including some from the SGEV affected herd in this study and some animals from all goat herds located within 10 km of the affected herd. Results confirmed that 5.1% of these adult goats had been exposed to a virus within the TBEV family [15]. In 2008, another goat herd, located in the same area as the affected herd described, showed similar clinical signs and 8 of 20 goats died, although a definitive diagnosis was not made (Official Veterinarians, personal communication). Furthermore, a study of wild ruminants in the region demonstrated a seroprevalence of 10.5%, and clinical disease was observed in two Cantabrian chamois (*Rupicapra pyrenaica parva*) [16]. These studies showed that viruses from the TBEV family are more widespread than the goat farm investigated here and that SGEV could have been present in Asturias for several years prior to the initial report. Asturias is a very mountainous region presenting natural barriers to easy movement of terrestrial animals between the fertile valleys. Therefore, the translocation of the goats may explain the severity of the lesions found, described only in unvaccinated goat kids [9,11], as the animals probably had no previous exposure to SGEV or related viruses prior to arriving in this now endemic region. 

The immunized goat (No. 3) in this study had cellular inflammatory lesions of even greater severity than the two non-immunized goats and this may be due to the higher cell-mediated specific immune response present in vaccinated animals in flavivirus infection [17]. Other possibilities to explain the severity of lesions and high incidence of clinical disease would be a co-infection with *Anaplasma phagocytophilum*, the agent of tick-born fever, as described previously [18]. However, no clinical signs suggestive of this co-infection were observed and there are no reports of clinical anaplasmosis in the region. Vaccination of goats against LIV using a commercially available vaccine has been shown to confer highly effective protection against experimental challenge with SGEV that was probably mediated by IgG [10]. However, vaccination failed to protect the goats in this natural outbreak, possibly because the disease was established already in the herd or, more likely, due to the lack of a second vaccination as this is required in species other than sheep [19].

The clinical signs and histological lesions observed in these goats naturally infected with SGEV resembled closely the morphology and distribution caused by LIV in sheep [6,20,21], including the greater involvement of the brainstem, cerebellum and proximal cervical spinal cord with the cerebral cortex relatively less affected [21]. However, whilst sheep showed more severe lesions in the ventral horns of the spinal cord [21], these goats presented with diffuse lesions affecting both the ventral and dorsal horns. Despite lesions having been reported in the hippocampus in lambs experimentally infected with LIV [22], the goats in this study (and those infected experimentally in a previous study [10]) were devoid of any lesion in this area and the mechanism and relevance of this difference is unknown. However, the high dose of LIV used for experimental infections and the longer post-infection survival time (21 days) may explain the presence of lesions in the hippocampus of lambs [22].

Severity and presentation of clinical signs in LI disease are related directly to the extent of damage to neurons [22,23], with neuropathological changes in moribund sheep most marked in the Purkinje cells, neurons of the dorsal motor nucleus of the vagus nerve and vestibular nuclei, and the ventral horns of the spinal cord [23]. This is in agreement with most of the areas affected in the three goats examined in this study, all of which developed severe neurological clinical signs. The distribution of viral antigens in goats Nos. 1 and 2 was restricted to the cytoplasm of neurons in several brain areas. However, only a small number of neurons were immunolabelled in any one particular area and these were not associated with inflammatory foci or inflammatory cells. LIV has a selective tropism for neurons, especially the cerebellar Purkinje cells which frequently show strong cytoplasmic labelling for LIV antigens [22,24]. In the present study, viral antigen within Purkinje cells was found only in goat No. 2, and of the two unvaccinated animals, it had the most severe lesions along with the most widespread immunolabelling. Differences in LIV immunolabelling have been reported previously in sheep [25]; only two of five sheep naturally infected with LIV showed immunolabelling in the brain. In vaccinated sheep that were intra-cerebrally challenged the cell-mediated immune reaction and perivascular cuffs were all more severe than in non-vaccinated animals and they also showed less labelling of LIV antigen [17]. This may explain the lack of immunolabelling and a greater cellular inflammatory response in samples from goat No. 3, as it had been vaccinated three months previously. Vaccination would have helped limit virus replication in response to natural infection due to a robust inflammatory response. However, as the vaccination was not fully protective, the ensuing robust cellular immune response was probably responsible for the severity of the lesions and, ultimately, the death of the animal. Similarly, fully vaccinated goats challenged with SGEV did not show any immunolabelling of viral antigen [10], although, in those cases, the cellular inflammatory reaction observed was very low and may have been due to a robust humoral response. Survival time post-infection may also influence the amount of immunolabelling as no labelling of SGEV was found in any brain section of goats challenged experimentally, although the virus had likely been eliminated prior to the day of sacrifice at 13–28 days post-infection [10]. In hamsters experimentally infected with West Nile virus (also genus *Flavivirus*), antigen presence was studied over time in the central nervous system using IHC [26]. West Nile virus antigen was not detected in the brain during the first five days after inoculation. On day 6, positive immunolabelling was observed in neurons in the basal ganglia and the brainstem; by day 7, in the cerebellar cortex and other regions of the brain; and after day 8, the amount of immunolabelling decreased. Goats Nos. 1 and 2 died one week after the onset of clinical signs. However, goat No. 3 died only 3 days after the onset of clinical signs, and although we were unable to detect SGEV by IHC in this case, the virus must have replicated in the brain for histological lesions and clinical neurological disease to have occurred. Therefore, the lack of detection of SGEV by IHC (viral antigen) in this animal may have been due to neutralizing antibodies from vaccination which, although not totally protective, could have somehow reduced the level of SGEV to below the detectable limit of the IHC method used. Although variation in susceptibility to infection with different strains of LIV has not been reported in previous studies in lambs [22], differences in immunolabelling due to variations in virus strain cannot be ruled out.

## 5. Conclusions

The likelihood that LI-causing viruses may spread from index cases depends on the level of viraemia present in the host, the presence of an appropriate species of tick, and the capacity of the latter to transmit the virus [27]. Even if goats are found not to be efficient maintenance hosts for SGEV, excretion of the virus in milk destined for human consumption could be a hazard to public health [9]. Tick monitoring and control schemes, in combination with vaccination of farm species capable of developing a significant viraemia, should decrease the incidence of LI-causing viruses in this area, where currently no goat herds are present.

SGEV should be considered a significant pathogen of goats that results in severe neurological clinical disease and high mortality. This study showed that the best samples for diagnosing LI caused by SGEV in goats by histopathology and IHC were the cervical spinal cord, brainstem and cerebellar cortex, whereas cerebral cortex gave inconsistent results. The use of histology and IHC, as well as molecular techniques to identify definitively the specific causal virus, will increase the diagnostic sensitivity for this disease.

## Figures and Tables

**Table 1:** Distribution and severity of histological lesions and presence of viral antigen in the brains and proximal cervical spinal cords of goats clinically affected by Spanish goat encephalitis virus (SGEV).

Goat No.	Anatomical Location							
	Spinalcord	Medulla Oblongata	Pons	Cerebellum		Thalamus/ Hypothalamus	Midbrain	Cerebral Cortex
				Peduncles	Cortex			
1 Lesions IHC	++ -	++ +	++ +	++ -	+++ -	++ -	++ -	+ -
2 Lesions IHC	+++ +	+++ +	+++ +	++ +	+++ +	+++ +	++ +	+ -
3 Lesions IHC	++ -	++ -	+++ -	++++ -	+++ -	++ -	++ -	+ -

Severity of lesions which consisted of perivascular cuffs, gliosis and neuronal necrosis. -: no lesion, +: mild, ++: moderate, +++: severe; ++++: very severe. IHC = Immunolabelling of SGEV virus using an Avidin-Biotin complex technique: -: no immunolabelling, +: positive immunolabelling.

## Data Availability

The data that support the findings of this study are available from the corresponding author upon reasonable request.
